# Draft Genome Sequence of the Strain Francisella tularensis subsp. *mediasiatica* 240, Isolated in Kazakhstan

**DOI:** 10.1128/MRA.00766-20

**Published:** 2020-08-27

**Authors:** Marat Kuibagarov, Alma Kairzhanova, Gilles Vergnaud, Asylulan Amirgazin, Larissa Lukhnova, Uinkul Izbanova, Yerlan Ramankulov, Alexandr Shevtsov

**Affiliations:** a National Center for Biotechnology, Nur-Sultan, Kazakhstan; b Institute for Integrative Biology of the Cell (I2BC), Université Paris-Saclay, Gif-sur-Yvette, France; c Masgut Aykimbayev Kazakh Scientific Center of Quarantine and Zoonotic Diseases, Almaty, Kazakhstan; d School of Science and Technology, Nazarbayev University, Nur-Sultan, Kazakhstan; University of Maryland School of Medicine

## Abstract

Francisella tularensis subsp. *mediasiatica* is the least studied among the four F. tularensis subspecies. We present here the genome data of F. tularensis subsp*. mediasiatica* 240, isolated in the southern region of Kazakhstan.

## ANNOUNCEMENT

Tularemia is a zoonotic natural focal infection caused by Francisella tularensis. Currently, four subspecies of F. tularensis are recognized, differing in virulence and geographical distribution. F. tularensis subsp. *tularensis* (type A) is common in North America. It is the most virulent subspecies for humans. The two subtypes A.I and A.II also differ in virulence (1). F. tularensis subsp*. holarctica* is the second most virulent for humans and is distributed in the Northern Hemisphere (2). F. tularensis subsp. *novicida*, described in North America and Australia, causes sporadic opportunistic infections in immunosuppressed patients (3, 4). F. tularensis subsp. *mediasiatica* remains the least-studied subspecies. For a long time, it was assumed that its distribution area was limited to Central Asia (Kazakhstan and Turkmenistan), but it was recently recovered in southern Siberia (5). No human infection caused by F. tularensis subsp. *mediasiatica* has been reported so far. Experiments on model animals indicate a virulence of F. tularensis subsp. *mediasiatica* intermediate between that of F. tularensis subsp. *tularensis* and F. tularensis subsp. *holarctica* (5).

The genetic diversity of F. tularensis subsp. *mediasiatica* is poorly known. In this article, we present the genome sequence of strain F. tularensis subsp. *mediasiatica* 240, isolated in 1982 from ticks in the southern region of Kazakhstan. The strain was isolated using direct plating of homogenized sample onto coagulated chicken egg yolk. The inoculations were kept under aerobic conditions at 37°C for 120 h, and typical colonies were subjected to reseeding and further typing. After identification, the strain was stored in a lyophilized state. Before the study, the lyophilized strain was suspended in 0.9% NaCl, plated onto petri dishes with FT agar including vitamins and mineral additives (FBIS SRCAMB, Obolensk, Russia) (5, 6), and cultured under aerobic conditions at 37°C for 72 h. A single colony was subcultured on a petri dish with FT agar and incubated at 37°C for 72 h. The bacterial mass was collected and suspended in 0.9% NaCl. The bacterial suspension was inactivated by adding a thimerosal solution (T5125, Sigma-Aldrich) to a concentration of 0.01% and incubated at 56°C for 30 min.

**FIG 1:**
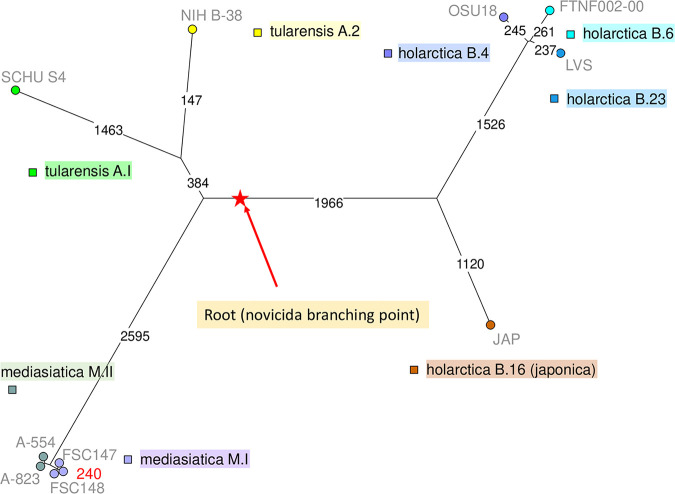
Maximum parsimony tree of whole-genome single nucleotide polymorphism (SNP) data. Whole-genome sequencing (WGS) data from all available F. tularensis subsp. *mediasiatica* strains, including Kazakhstan strain 240 (indicated in red), and from selected strains representing the main sublineages of F. tularensis subsp. *tularensis* and F. tularensis subsp. *holartica* were mapped onto reference genome SCHU S4 (assembly accession no. GCA_000008985), as previously described (5). In total, 10,953 SNPs were called; the tree size is 11,027 bp (homoplasia, 0.67%). Branch length of the longer branches is indicated. Branches of 70 and 44 SNPs lead to lineages M.I and M.II (Siberian strains), respectively. The red star indicates the branching point toward F. tularensis subsp. *novicida*, which can be considered an outgroup with respect to the three other subspecies.

DNA was isolated using a DNA minikit (Qiagen, Hilden, Germany). Preparation of the sequencing libraries was carried out using the Nextera XT DNA library prep kit (Illumina, San Diego, CA, USA). Sequencing was performed using the MiSeq system with the MiSeq reagent kit v3 (600 cycles, 2 × 300 bp). In total, 780,074 sequencing reads were obtained. The reads were trimmed using Seqtk v1.3 (7) up to a quality (Q) value of Q30 and *de novo* assembled with Skesa v2.3.0 (8) (all software was used with default parameters except when stated otherwise). Assembly quality assessment was performed using QUAST v5.0.2 software (9). The draft genome assembly totaled 1,791,721 bp, with 75 contigs, an average coverage of 71×, an *N*_50_ value of 35,408 bp, and a GC content of 32.33%. Genome annotation was performed using the NCBI Prokaryotic Genome Annotation Pipeline (PGAP v4.11) (10, 11). Totals of 265 pseudogenes and 1,525 genes were predicted, of which 1,483 are protein coding genes and 42 are RNA coding genes. The genetic diversity described within F. tularensis subsp. *mediasiatica* is very limited compared to the genetic diversity reported in the other F. tularensis subspecies (Fig. 1). Strain 240 belongs to F. tularensis subsp. *mediasiatica* subtype M.I (Fig. 1).

### Data availability.

This whole-genome shotgun project has been deposited in DDBJ/EMBL/GenBank under the accession no. JABWGW000000000. The version described in this paper is the first version, JABWGW010000000. The raw data from BioProject PRJNA639508 were submitted to the NCBI SRA under experiment accession no. SRR12015651.
